# Accuracy of erythrogram and serum ferritin for the maternal anemia diagnosis (AMA): a phase 3 diagnostic study on prediction of the therapeutic responsiveness to oral iron in pregnancy

**DOI:** 10.1186/1471-2393-13-13

**Published:** 2013-01-16

**Authors:** Cristiane Campello Bresani, Maria Cynthia Braga, Daniel Falcão Felisberto, Carlos Eduardo Lopes Tavares-de-Melo, Debora Bresani Salvi, Malaquias Batista-Filho

**Affiliations:** 1 Nutrition Research Group at Instituto de Medicina Integral Prof Fernando Figueira – IMIP, Rua dos Coelhos, 300, Boa Vista, Recife, PE CEP: 50.070-550, Brazil; 2 Instituto Nacional do Seguro Social/Ministério da Previdência Social – INSS/MPS, Av Jorn Mário Melo, 343, Santo Amaro, Recife, PE CEP: 50.040-010, Brazil; 3 Postgraduate Program in Maternal and Child Health of IMIP, Rua dos Coelhos, 300, Boa Vista, Recife, PE CEP: 50.070-550, Brazil; 4 Postgraduate Program in Public Health at Centro de Pesquisas Aggeu Magalhães – Fundação Oswaldo Cruz – CPQAM/FIOCRUZ, Av. Professor Moraes Rego, s/n - Campus da UFPE - Cidade Universitária, Recife, PE CEP: 50.670-420, Brazil; 5 Faculdade Pernambucana de Saúde – FPS, Av. Jean Emile Favre, 422 Imbiribeira, Recife, PE CEP: 51.200-060, Brazil

**Keywords:** Anemia, Iron-deficiency, Diagnosis, Pregnancy, Erythrocyte indices, Ferritins, Sensitivity, Specificity, Adverse effects, Patient compliance, Ferrous sulfate

## Abstract

**Background:**

Pregnancy anemia remains as a public health problem, since the official reports in the 70’s. To guide the treatment of iron-deficiency anemia in pregnancy, the haemoglobin concentration is the most used test in spite of its low accuracy, and serum ferritin is the most reliable test, although its cutoff point remains an issue.

**Methods/design:**

The aim of this protocol is to verify the accuracy of erythrocyte indices and serum ferritin (studied tests) for the diagnosis of functional iron-deficiency in pregnancy using the iron-therapy responsiveness as the gold-standard. This is an ongoing phase III accuracy study initiated in August 2011 and to be concluded in April 2013. The subjects are anemic pregnant women (haemoglobin concentration < 11.0 g/dL) attended at a low-risk prenatal care center in the Northeast of Brazil. The sample size (*n* 278) was calculated to estimate sensitivity of 90% and 80% of specificity with relative error of 10% and power of 95%. This study has a prospective design with a before-after intervention of 80 mg of daily oral iron during 90 days and will be analyzed as a delayed-type cross-sectional study. Women at the second trimester of pregnancy are being evaluated with clinical and laboratorial examinations at the enrollment and monthly. The ‘responsiveness to therapeutic test with oral iron’ (gold-standard) was defined to an increase of at least 0.55 Z-score in haemoglobin after 4 weeks of treatment and a total dose of 1200 mg of iron. At the study conclusion, sensitivities, specificities, predictive values, likelihood ratios and areas under the ROC (Receiver Operating Characteristic) curves of serum ferritin and erythrocyte indices (red blood cell count, haematocrit, haemoglobin concentration, mean corpuscular volume, mean corpuscular haemoglobin, mean corpuscular haemoglobin concentration, red blood cell distribution width, reticulocyte count) will be tested. The compliance and adverse effects are considered confounding variables, since they are the main obstacles for the iron-therapy responsiveness.

**Discussion:**

This study protocol shows a new approach on iron-deficiency anemia in pregnancy from a functional point of view that could bring some insights about the diagnostic misclassifications arising from the dynamic physiologic changes during the gestational cycle.

**Trial registration:**

WHO International Clinical Trials Registry Platform U1111-1123-2605.

## Background

Iron-deficiency anemia is recognized as the nutritional deficiency of highest prevalence in the world, reaching all continents and geo-economic blocks [1,2]. This is a serious problem in the gestation-puerperal period since the maternal anemia is associated with several adverse perinatal outcomes, such as prematurity, low birth weight and rates of maternal and perinatal mortality [3-9]. For instance, the relationship between maternal anemia and perinatal mortality contributed with 56% of 800,000 deaths attributed to iron-deficiency anemia globally in 2000 [9]. Hence, universal supplementation of iron during pregnancy has been widely recommended as a health policy from the 70´s [1,10]. However, the prevalence of gestational anemia remains 50% globally and 20% in the Americas since then [1,2] which points to the failure of this measure, despite the effectiveness of the iron supplementation programs in the general population has been estimated to about 70% [1].

Apart of the concerning issues to the implementation degree and quality of the programs to control anemia, the main limiting factor of the effectiveness and efficacy of the interventions with iron supplements would be the poor compliance to therapy, which can achieve figures up to 70% at pregnancy [11,12] and is associated with the frequent occurrence (20-70%) of gastrointestinal adverse effects [13-18]. Taking into account that the therapeutic success depends on the total dose of iron ingested throughout the treatment [14,18], higher cure rates of anemia should be observed in the controlled context of clinical trials, whereas the therapeutic compliance would be maximized, however, these rates are around 50% [19,20]. Therefore, factors not related to the therapeutic dose of iron may be contributing to these modest results, among them could be mentioned the criteria used to indicate and evaluate the iron-therapy during pregnancy [1,2,10].

The haemoglobin concentration (Hb) has been considered as a *proxy* of iron deficiency and widely recommended as a criterion for the indication of iron-therapy in pregnant women, particularly in location with few resources [1]. The Hb cutoff point of 11.0 g/dL for pregnancy was defined from North American and European reference populations by the Gaussian paradigm: less than 2 standard deviations below the mean reference [1,21,22]. This is a theoretical statistic definition that assumes prevalence of 5% for all disorders and might not be suitable for all epidemiologic or clinical settings [23]. Indeed, the 70’s guideline of the World Health Organization (WHO) considered that the Hb cutoff points defined from population distributions could be an oversimplification [10]; and some studies have observing that Hb and other erythrocyte indices present low correlation with the body iron reserves in pregnant women (sensitivity and specificity around 60%) [24-29]. Thus, could be considered that diagnostic misclassifications of iron-deficiency anemia may be another limiting factor of the iron-therapy responsiveness in this life cycle phase.

In fact, the cross-sectional and longitudinal assessment of the haematological profile during pregnancy is troublesome due to increased iron requirements beside the haemodilution, a physiological phenomenon in which occur an increase of 50% in the plasma volume *versus* 30% in the erythrocyte mass [1,21]. This phenomenon is responsible for the U-shaped curve that Hb and haematocrit levels develop in pregnant women with nadir between the 24th and 28th weeks of pregnancy [24,30-32], which explains the “physiological anemia” in which the erythrocyte mass remains normal in relation to the effective body weight [1,7]. Based on this rationale, Beaton and McCabe (1999) developed the Hb Z-score methodology to correct its values according to the mean expected for the gestational week [33]. An alternative to a more accurate diagnosis of iron deficiency would be the dosage of biomarkers of iron metabolism, however, these are also influenced by gestational physiology and many have not been standardized to be used at pregnancy [1,34]. The serum ferritin remains as the biomarker that best correlates with the iron content in the bone marrow of pregnant women [24-27], but there is no consensus as to its cut-off point [1,34].

These observations indicate that the accurate diagnosis of iron-deficiency anemia during pregnancy is problematic. The current diagnostic criteria with steady cutoff points based on Gaussian definition of normality does not reflect the functional conceptions of the anemia and of the iron deficiency at pregnancy, respectively, as the status of insufficient circulating haemoglobin for oxygen transport required by gestational metabolism and as the inadequate iron supply to achieve this demand [1]. So, the hypothesis of AMA study is that the pre-treatment values of Hb and other erythrocyte indices have low power to predict the functional iron-deficiency in pregnancy and to discriminate iron-sufficient from iron-deficient pregnant women (potentially responsive to iron-therapy). It is expected that the serum ferritin presents higher accuracy parameters than erythrocyte indices.

### Rationale for AMA study - phase III diagnostic accuracy study

Starting from the assumptions that erythrocyte indices have low accuracy for the diagnosis of iron deficiency in pregnant women in accuracy phase II studies [24-29], the AMA study was initiated. As an accuracy phase III study, it is being conducted in a population under a higher risk of iron deficiency [23]. Thereby, this study will evaluate and compare the accuracy of Hb and other erythrocyte indices and of serum ferritin for the diagnosis of functional iron deficiency in anemic pregnant women (Hb < 11.0 g/dL), as pregnant women with Hb > 11.0 g/dL from the same location have very low rates of iron-deficiency [35].

In the absence of a gold standard to define functionally iron deficiency in pregnancy, was established as the reference standard the haematological response to oral iron-therapy, which is considered a reliable and low-cost alternative for the confirmation of anemia due to iron deficiency [1]. In this protocol, this approach considers the rationale of therapeutic definition of normality (therapeutic diagnostic) that employs the response to specific treatments in disease-target markers as the parameter to define diagnostic criteria [23]. This functional rationale is suitable for the pregnancy period because the dynamic physiologic changes on the erythrocyte indices and biomarkers of iron metabolism [30-34].

Taking into account that adverse effects and poor therapeutic compliance are the main limiting of intake of an effective dose of iron they were pre-established as confounding variables. Whereas the physiological fluctuation of Hb at each gestational week is a factor which can be bias the evaluation of the haematological response to iron-therapy [33], the pre and post treatment Hb values will be transformed into Z-scores for purposes to measure the outcome.

## Methods/design

### Study aim and objectives

The aim of this study is to describe and to compare, at the practice setting of prenatal care, the pragmatic utility of serum ferritin and each single test on erythrogram to discriminate iron-sufficient from iron-deficient pregnant women who will benefit from iron therapy to achieve the improvement of their anemia.

The *primary objective* of this study is to analyze the accuracy (sensitivity, specificity, predictive values, likelihood ratios and Receiver Operating Characteristic curves) of Hb and other erythrocyte indices and of serum ferritin (studied tests) to predict the ‘responsiveness to therapeutic test with oral iron’ (gold-standard test) in pregnant women pre-classified as anemic (Hb < 11.0 g/dL).

As a *secondary objectives* are proposed to compare the results of evaluation of the ‘responsiveness to therapeutic test with oral iron’ using absolute values and Z-scores of Hb; and to describe the frequency of the therapeutic compliance and gastrointestinal adverse effects, as well as their association with the dose of iron intake and with the therapeutic response.

### Study design

This study deals with a diagnostic validation of the pre-treatment values of erythrocyte indices and serum ferritin (studied tests) in relation to the gold standard ‘responsiveness to therapeutic test with oral iron’ in women with low-risk singleton pregnancy. The design is classified as phase III as these tests already have been used in clinical practice and will be evaluated in anemic pregnant women [23], i.e., a population under risk of iron deficiency (target disease) with formal indication of oral iron therapy in accordance with the criterion adopted by WHO (Hb < 11.0 g/dL) [1]. The direction of the study is prospective (delayed-type cross-sectional study) with the purpose of observing the variation in the Hb values after a period of at least 4 weeks of a ‘before-after’ intervention with 80 mg of daily oral iron and to define the final diagnosis of the functional iron deficiency [36]. The measurement of target disease (iron deficiency) by the gold-standard (‘responsiveness to therapeutic test with oral iron’) has been carried out on all participants, such as post-treatment Hb has been assessed independently of the results of the studied tests (pre-treatment erythrocyte indices and serum ferritin). The final diagnosis of the functional iron deficiency will be blinded in relation to the tests results carried out at time-zero since it will be calculated by the Hb Z-scores methodology at the conclusion of the study.

The protocol is registered as a single-arm clinical trial in the Brazilian Clinical Trials Registry (REBEC) at the Ministry of Health of Brazil and in the WHO International Clinical Trials Registry Platform (U1111-1123-2605).

### Study setting and period

This study is set in the prenatal care center of *Instituto de Medicina Integral Prof Fernando Figueira* (IMIP) at a large urban center in the Northeast of Brazil. IMIP is a regional tertiary hospital with reference in maternal-child health which serves primarily for high-risk pregnant women; however, around 600 low-risk pregnant women are attended monthly. Data collection was initiated in August 2011 in a pilot study phase in which was concluded in October 2011. The conclusion of this study is scheduled for April 2013.

### Participants/eligibility criteria

The participants are 18-35 years old women with a low-risk singleton pregnancy. The inclusion criteria are Hb values ≥ 7.0 and < 11.0 g/dL and the gestational age between 12 and 32 weeks. Pregnant women are being excluded if they have a history of hypersensitivity or intolerance to ferrous sulfate, mental deficits or disorders that cannot correctly follow the prescription; tobacco, alcohol or other drugs use; prior diagnosis of another cause of anemia; or at the time of inclusion present active infectious disease (positive serology for Human Immunodeficiency Virus or syphilis, leukocytosis or leukocyturia with positive urine culture).

### Recruitment, procedures and instruments

**Figure 1:**
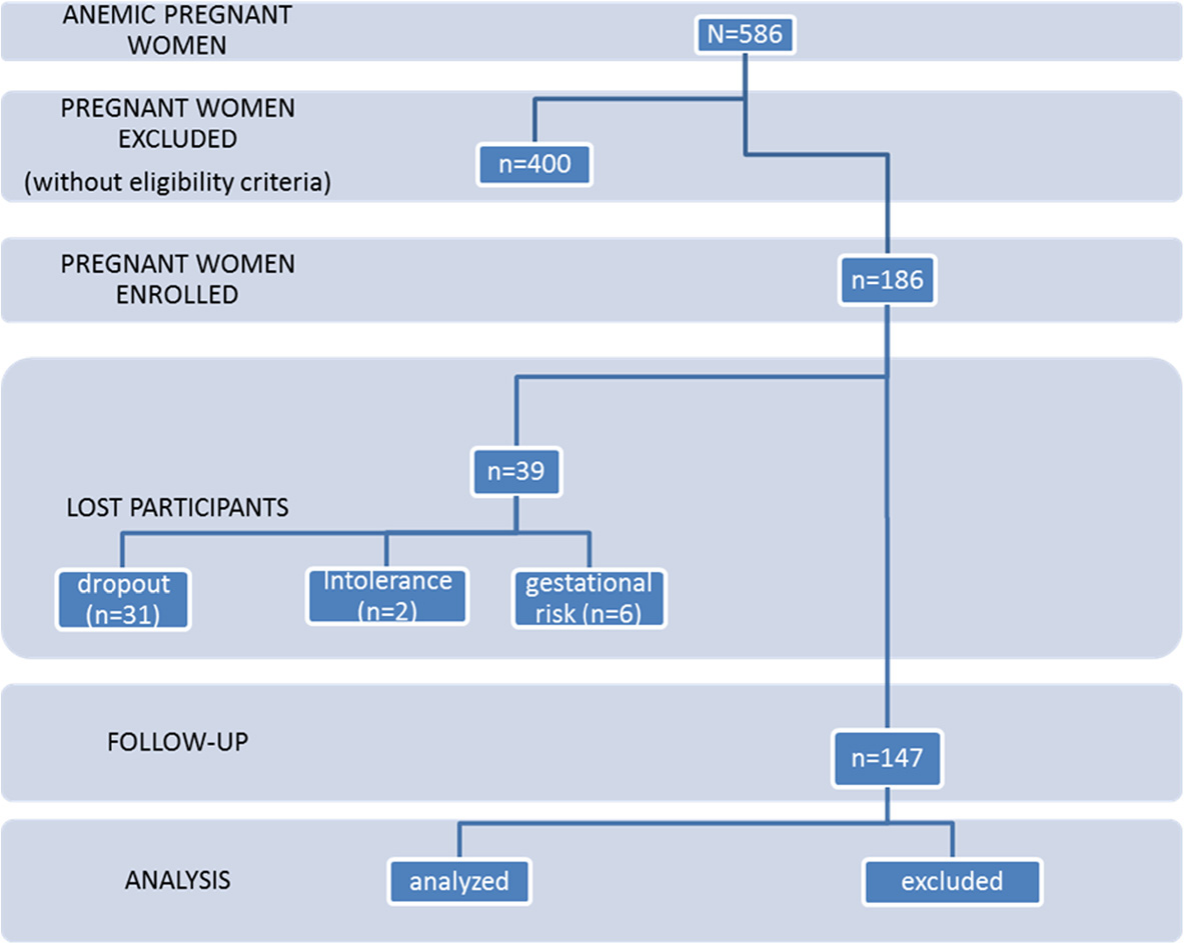
Flowchart of the participant enrolled in the AMA study until august 2012.

The recruitment procedure is a consecutive series of patients whose prenatal routine exams show anemia (Hb < 11.0 g/dL) and encounters all other eligibility criteria (Figure 1). The pregnant women are then invited to participate in the study at the time of the prenatal consultation to then clarify about the issue. After reading and signing the written informed consent form, an initial consultation begins (C_0_) with a structured questionnaire and the anthropometric variables measurement (weight and height). At this moment, the prescription for the drug intervention proceeds under the proper guidance and then the pregnant woman is guided to perform the initial laboratory tests (complete blood count, reticulocyte count and serum ferritin).

The pregnant women are measured and weighed barefooted, without any objects in their hands or in their pockets on a calibrated electronic scale. It is requested that all pregnant women attend the laboratory in the morning within 8–12 hours of fasting to collect blood samples. The blood samples are collected and properly identified by the process of collecting in the routine service, through venipuncture in the antecubital fold. The erythrograms are analyzed using flow cytometry and absorbance of an automatic hematological analyzer (ABX Pentra DF120 Horiba brand) calibrated daily, and are complemented with a microscopic reading of smears stained with a panoptic dye to morphologically study of the cells. The reticulocyte count is performed by a manual method by reading of smears stained with brilliant cresyl blue dye. The serum ferritin is measured by chemiluminescence immunoassay in blood sample collected in dry tube, using the same kit and following the calibration according to the international standards of WHO.

All non-laboratorial variables are obtained using a standardized form developed specifically for the research. The laboratory data are recovered directly by a computerized system of results generated electronically by the automated equipment of biological analysis. The laboratory tests of the pilot study (*n* 23) were performed by the laboratory of the institution. After the conclusion of the pilot study (October 2011), for operational reasons, the laboratorial analysis service was outsourced to an external laboratory. Both laboratories have governmental certification and follow standardized operational norms.

### Intervention

The treatment consists of two daily doses of 109 mg of ferrous sulfate in the form of pills with 40 mg of elemental iron (Hematofer®, Prati Donaduzzi & Cia LTDA). Three blisters with 20 pills are given at the enrollment (C_0_) and at the two monthly revaluations (C_1,_ C_2_). The pregnant women are oriented at each consultation to ingest the medication with a glass of drinking water, 30 minutes before a meal, and to preserve the non-consumed pills in the blisters. The safety profile for the use of ferrous sulfate at pregnancy is satisfactory; there were no reports of fetal damage or severe maternal adverse events [19,20]. The dose of 80 mg/daily of elemental iron was adopted due to the set of evidences not found any additional efficacy with higher daily doses [14,19].

### Follow-up and withdrawal criteria

The prescribed treatment provides a follow-up period of 90 days. The follow-up is stopped before this period in case of evolution to high-risk pregnancy, genital bleeding, childbirth delivery, drop out of treatment, use of another type of iron supplement, drug intolerance, cure or aggravation of anemia. The participants are evaluated monthly (C_1,_ C_2,_ C_3_), and information about gastrointestinal symptoms and therapeutic compliance is collected by the standardized form, such a venous blood sample to obtain Hb is prompted. Pregnant women who present drug intolerance, severe anemia (Hb < 7.0 g/dL) or Hb values drop more than 1.0 g/dL during the follow-up are referred to an individualized conduct. Those who present Hb > 11.0 g/dL before 90 days of the overall follow-up will begin to use supplemental doses of oral iron (40 mg/daily).

### Studied tests (predictive variables)

The initial values of the erythrocyte indices and serum ferritin will be tested as predictors of presence of the functional iron deficiency at the following cutoff points suggested by WHO and Centers for Disease Control and Prevention (CDC) on pregnant or childbearing age women (when there is no specific report for pregnant women): red blood cells count < 3.8 10^12^ cells/L; Hb < 10.5 g/dL (suggested for the 2nd trimester of pregnancy); haematocrit < 32.0% (suggested for the 2nd trimester of pregnancy); mean corpuscular volume (MCV) < 81.0 fL; mean corpuscular haemoglobin (MCH) < 26.0 pg; mean corpuscular haemoglobin concentration (MCHC) < 32.0 g/dL; red blood cells distribution width (RDW) > 14.0%; reticulocyte count < 1.0%; serum ferritin < 12.0 ng/mL [1,21,22].

### Gold-standard test (outcome variable)

The final diagnosis of iron deficiency will be set individually on the basis of the presence (case) or absence (non-case) of the ‘responsiveness to therapeutic test with oral iron’, starting from assumptions that, in the case of iron deficiency in pregnancy, a functional definition would be more appropriate [37] and the haematologic response to the treatment is considered as a reliable indicator of functional iron need [1]. This is one of the alternative approaches in the definition of the gold-standard which is based on the follow up of the clinical course during a suitable predefined period under a therapeutic intervention [36].

To measure this outcome, the physiological variability of Hb throughout the pregnancy was taken into consideration, because it distorts the interpretation of longitudinal trends of Hb absolute values [33]. Thus, on the rationale basis developed by Beaton and McCabe in 1999, the pre and post treatment Hb values of each pregnant woman will be adjusted to the gestational week through the Z-score methodology [33]. This methodology quantifies the difference (in standard deviation units-SD) between an observed Hb value and the gestational week’s reference mean from a reference distribution curve [33]. Due to the lack of a Brazilian reference curve will be used the curve reported from iron-supplemented healthy women from Europe and North-America which was employed by Beaton and McCabe [21,22,33]. The individual calculation of the Z-score is carried out according to the following mathematical formula:

$$\mathrm{Hb}\;\text{Z}-\text{score}=\frac{\left(\mathrm{Hb}\;\mathrm{value}\;\mathrm{observed}-\mathrm{mean}\;\mathrm{of}\;\mathrm{Hb}\;\exp\mathrm{ected}\right)}{\mathrm{SD}\;\mathrm{of}\;\mathrm{the}\;\mathrm{reference}\;\mathrm{population}}$$

It was determined as a final diagnosis of functional iron deficiency the partial therapeutic responsiveness by increase of at least 0.55 Hb Z-score after a minimum of 4 weeks treatment and an intake of at least 1200 mg of elemental iron as a total dose, based on the following assumptions:

– An increase of 1 g/dL on the Hb after 30 to 60 days of oral iron therapy is a reliable indicator of iron deficiency in individuals or populations [1].

– The haematological improvement depends on the total dose of iron intake considering that the dose of 1200 mg of elemental iron is responsible for most of the effect on the Hb values [14].

– The standard deviation of the Hb distribution in populations of pregnant women is 0.9 g/dL, independently of gestational age [33].

– By mathematics definition, 1 Z-score corresponds to 1 SD, that is, equal to 0.9 g/dL of Hb at pregnancy. Therefore, the difference of 0.55 SD between the post and pre-treatment Hb Z-scores represents a relative increase of 0.5 g/dL (0.55 × 0.9 g/dL) in the Hb *status* of a pregnant woman.

### Confounding variables (secondary outcomes)

Treatment compliance and adverse effects are being evaluated every 30 days and recorded in the pregnant women´s individual form with a goal to reduce losses and ensure the intake of the effective total dose of iron with minimal symptomatic discomfort for the pregnant women. Women who do not achieve the minimum adherence (intake of 30 pills = 1200 mg of elemental iron) will not be evaluated for the ‘responsiveness to therapeutic test with oral iron’. The frequency of the good adherence to the treatment will be reported as an intake at least 75% of the prescribed monthly pills, according to pregnant women’s information and pill counting. This percentage was determined on the basis of the proportion of the monthly treatment prescribed that corresponds to the total monthly dose of 1800 mg of elemental iron, which is considered responsible for almost the entire effect in Hb levels according to Ekström *et al.* (2002) [14].

According to pregnant women´s information the presence of the adverse effects was defined as the appearance of the following symptoms after the beginning of the intervention: abdominal pain/abdominal cramps, diarrhea (increasing number of evacuations or reduction of the stools consistency), constipation (reducing number of evacuations or hardening of stools), nausea, vomiting and heartburn (epygastric burning or heartburn) [38]. The women who developed symptoms related to the medication are orientated case by case to ingest the pills along with the meals or temporarily reduce the dosage for a pill a day.

The following *co-variables* are being collected for the sample characterization: age, city of residence, education level, socioeconomic class (economic classification criteria Brazil 2010) [39], marital status; anthropometric classification by the body mass index (Atalah *et al.* standard) [40], number of pregnancies, births or miscarriages and last inter-gestational interval. The gestational age is being defined as complete weeks from the date of the last menstrual period and confirmed by ultrasound estimation. In pregnant women who do not remember their last menstrual period, the gestational age will be based solely on the ultrasound examination.

### Statistical issues

#### Sample size

The sample size (43 cases and 97 non-cases) was calculated to estimate 90% of sensitivity and 80% of specificity, with relative error of 10% and the power of 95%. Considering cure rates of 50% and 30% of losses or poor adherence to treatment [38,41], 278 anemic pregnant women should be recruited.

#### Operational and data analysis

The data are being released with dual input and processed in the EPIINFO3.4.2 program. The analytical design is as a delayed-type cross-sectional study, where at the end of the follow-up, the measured tests at time-zero (predictive variables) will be compared with the final diagnosis of the target-disease (outcome variable) to determine estimates of accuracy [36]. Thus, at the end, the proportions and its confidence intervals will be calculated for sensitivity, specificity, accuracy, predictive values, likelihood ratios and areas under the ROC curves of each erythrocyte index and serum ferritin against the presence or absence of the ‘responsiveness to therapeutic test with oral iron’. The significance level adopted will be 5%. The estimates will be made according to the 3 × 3 table for the results distribution from diagnostic accuracy phase III studies, which have cells to report unconcluded results (lost, not performed or indeterminate) of the gold standard and the studied tests [23]. This table enables to report the results unknown that could bias the measurement of accuracy at a clinical practice setting (Table 1).

**Table 1:** The table 3 × 3 to distribute the tests results on phase III accuracy studies

	**Reference standard ‘responsiveness to therapeutic test with oral iron’**			
**Diagnostic test result (erythrocyte indices or serum ferritin)**	**Iron deficiency present**	**Lost, not performed or indeterminate**	**Iron deficiency absent**	**Total**
Positive	*a*	*v*	*b*	
Lost, not performed or indeterminate	*w*	*x*	*y*	
Negative	*c*	*z*	*d*	
Total				

#### Pilot study and interim analysis

During the period of the pilot study 106 anemic pregnant women were identified, which 39 fulfilled the eligibility criteria with the acceptance of 100% to participate in the research. The loss percentage was 41% due to dropout (9), gestational risk (5) and drug intolerance (2). Low therapeutic adherence (intake of less than 75% of pills) was observed in 30.4% (7/23) and the most incidents of adverse effects were nausea (25.8%), heartburn (21.5%) and constipation (17.2%).

An interim analysis of collected data will be performed on 186 pregnant women enrolled until August 2012, whose follow-ups were completed in November 2012, to verify the number of cases and non-cases and estimate the additional time necessary to achieve the sample size.

### Ethical issues

This protocol follows the Ethical Principles for Medical Research Involving Human Subjects of the Declaration of Helsinki and the 196/96 resolution from the National Health Council of Brazil. This study has the ethical approval for the human experimentation by IMIP Research Ethics Committee under the number 2050–10. The participants are duly informed about the research explained by the team and are enrolled in the study after having assigned the written informed consent form.

### Financing

This trial was funded by the National Council for Scientific and Technological Development (CNPq) of Brazilian Government as a nested study to the cross-sectional multicenter survey ‘*Pregnant women nutritional status in the state of Pernambuco: methodological, epidemiological aspects and implications in pre-natal care’.*

## Discussion

Pregnancy is a period marked by singularities in the physiology of the body fluids and erythropoiesis, so both haemodilution and iron deficiency leads to anemia and this discrimination becomes difficult [21,34,37]. The protocol study AMA applies a new approach to the problem of diagnosis of iron-deficiency anemia in pregnancy, regarding to the design of the study and to the definition of the target disease. As a phase III study, the diagnostic accuracy of the tests will be evaluated in the practical context of prenatal care, where the contrast between the presence and the absence of iron deficiency is attenuated making it difficult to distinguish pregnant women who are or not potentially iron responsive [23,36]. Thus, this study design needs larger samples, which makes its use uncommon [36].

In the absence of a gold standard, the haematological response to oral iron was adopted as the reference standard in order to define iron deficiency in pregnancy under the functional point of view. This approach aims to evaluate the pragmatic usefulness of erythrogram and serum ferritin to identify pregnant women who would benefit from iron therapy to achieve the improvement of their anemia, which should be the theoretical purpose of the current guideline in recommending iron-therapy for all pregnant women with Hb < 11.0 g/dL [1]. However, the gold-standard ‘responsiveness to therapeutic test with oral iron’ is the main fragility of this protocol, because it is subordinate to the total dose of iron ingested, the effective absorption of iron ingested and the reliable measurement of haematological response [14,33]. Thus, the follow up on therapeutic compliance and adverse effects was incorporated into the protocol so it can be a guarantee the intake of the effective total dose of iron. With regard to the absorption of iron ingested, it was considered that it would not constitute an important limiting factor to the therapeutic response since it increases up to 40% during pregnancy [34]; furthermore the women are being guided to take the iron pills in the most effective way.

An important issue addressed in the protocol method is the physiologic fluctuation of the Hb absolute values which could bias the therapeutic efficacy measurements during the follow-up trial period [24-29]. Beaton and McCabe (1999) argued that, depending on the gestational week in which the treatment of anemia is initiated and concluded, variations observed in the Hb levels may be attributed to its physiological curve and could be confounded with the therapeutic effects. These authors reanalyzed 21 trials with oral iron in children and pregnant women and observed important differences between the outcomes assessed by Hb absolute values and by the Hb Z-scores [33]. Thus, in this protocol the post and pre-treatment difference in Hb values of each pregnant woman will be measured in the Z-scores, aiming to neutralize the variability intra-observation, resulting from physiological Hb curve of each woman, and the variability inter-observation, arising from the different gestational ages represented in the sample. Despite its appropriateness, the Z-score methodology has been scarcely used in clinical trials of treatments for iron-deficiency anemia in pregnancy [19]; for instance, Mumtaz *et al.* (2000) were the only one to apply the Hb Z-scores among 23 clinical trials reviewed in the most recent meta-analysis [19,42].

In addition to these methodological questions, this study found its own operational limitations of the routine at a prenatal service in our location, particularly regarding to the patients’ return for clinical follow-up and the effective performance of laboratorial exams. The excessive loss rate of the pilot study was overcome with strategies which have strengthened the follow-up and the laboratory analyzes. The percentage of adverse effects agreed with the literature, signaling the continuance of the study in order to ensure the effective dose of iron intake and the safety standard of the medication [11-17]. At the end of the study, we hope that the results will bring some clarification on the role of Hb and serum ferritin in the diagnosis of iron deficiency in pregnancy and that allows us to question the paradigm that *“the prevalence of iron deficiency anemia in a population is statistical rather than physiological concept”* (WHO, 2001) [1].

## Competing interests

The authors declare that they have no any financial competing interests as well as other potential conflicts of interest related to this study.

## Authors’ contributions

CCB, MCB and MB conceived and designed this protocol. CCB, DFF, CET and DBS are carrying out the acquisition of data. CCB is performing the study coordination. All authors read, critically revised and approved this manuscript.

## Pre-publication history

The pre-publication history for this paper can be accessed here:

http://www.biomedcentral.com/1471-2393/13/13/prepub
